# Exploring the Relationship Between Internet Use and Mental Health Among Older Adults in England: Longitudinal Observational Study

**DOI:** 10.2196/15683

**Published:** 2020-07-28

**Authors:** Sabrina Sze Man Lam, Stephen Jivraj, Shaun Scholes

**Affiliations:** 1 University College London London United Kingdom

**Keywords:** internet, socioeconomic factors, mental health, life satisfaction, depression, effect modifier

## Abstract

**Background:**

There is uncertainty about the impact of internet use on mental health in older adults. Moreover, there is very little known specifically about the impact of particular purposes of internet use.

**Objective:**

This study aims to investigate the longitudinal relationship between two distinct concepts of mental health with the frequency of internet use among older adults: the moderating role of socioeconomic position (SEP) and the association between specific purposes of internet use.

**Methods:**

Longitudinal fixed and random effects (27,507 person-years) models were fitted using waves 6-8 of the English Longitudinal Study of Ageing to examine the relationship between different aspects of internet use (frequency and purpose) and two mental health outcomes (depression and life satisfaction). The potential moderating effect of SEP on these associations was tested using interaction terms.

**Results:**

Infrequent internet use (monthly or less vs daily) was predictive of deteriorating life satisfaction (β=−0.512; *P*=.02) but not depression. Education and occupational class had a moderating effect on the association between frequency of internet use and mental health. The associations were stronger in the highest educational group in both depression (*P*=.09) and life satisfaction (*P*=.02), and in the highest occupational group in life satisfaction (*P*=.05) only. Using the internet for communication was associated with lower depression (β=−0.24; *P*=.002) and better life satisfaction (β=.97; *P*<.001), whereas those using the internet for information access had worse life satisfaction (β=−0.86; *P*<.001) compared with those who did not.

**Conclusions:**

Policies to improve mental health in older adults should encourage internet use, especially as a tool to aid communication.

## Introduction

As population aging brings major challenges for society and health care services, promoting healthy aging is important to prevent the burden and financial costs related to aging-associated diseases. Mental health is a crucial component of healthy aging. Poor mental health can profoundly affect the quality of life of older adults and is associated with an increased risk of mortality and morbidity [1,2].

Risk factors for poor mental health in older adults could be potentially impacted by internet use; maintaining and building social relationships through email or social networking sites may increase feelings of social support and connectedness and relieve feelings of isolation and loneliness [3-7]. The internet is used at least once every 3 months by an increasing proportion of the English population aged 50 years and older (63% and 51% of men and women, respectively, in 2010 compared with 84% and 78% of men and women, respectively, in 2016) [8]. However, internet use remains lower in older adults than in younger adults [9].

Evidence is mixed on the impact of internet use on mental health in older adults. Forsman and Nordmyr [10] reported a balanced positive association in a review of 18 quantitative and 14 qualitative studies but found that 40% of the outcomes they reviewed showed no association. Claims for positive impacts according to some researchers are often exaggerated because of training effects, inappropriate generalization of results, and misattribution of causality [11]. Training effects and limited generalizability are typically leveled in experimental studies where a small intervention group of selective participants is given support such as training to use the internet [12-14]. However, recent evidence suggests that internet use can improve mental health. Using data from the US Health and Retirement Study (HRS) over a 6-year period, Cotten et al [15] found that prior internet usage reduced the probability of depression by one-third between 2002 and 2008 in a sample of 3000 retired adults aged 50 years and older. Using data over a 10-year period, Lissitsa and Chachashvili-Bolotin [16] found that internet adoption among an Israeli sample of adults aged 65 years and older was associated with increased life satisfaction, with the strength of the estimated association being as strong as that found for being married.

To our knowledge, no literature has longitudinally examined the associations between internet usage and multiple subdimensions of mental health in the same sample. The dimensions of mental health include hedonic and eudemonic well-being [17]. According to Vanhoutte [18], hedonic well-being refers to pleasure maximization and suffering minimization, with the cognitive and affective aspects of well-being forming subdimensions of hedonic well-being. Cognitive well-being is an evaluative process in which an individual assesses the quality of their life; in contrast, affective well-being refers to positive and negative moods and emotions that are not simply the opposite of one another [18,19]. Eudemonic well-being is conceptually distinctive in that it refers to a sense of purpose or control [20]. Separating these dimensions is important (through, eg, separate measurement scales for life satisfaction, depression, and quality of life) because they capture varied aspects of mental health; these could, theoretically, be influenced by internet use in different ways. For example, Matthews and Nazroo [21] explored the cross-sectional relationship between internet use and several dimensions of mental health using data from persons aged 50 years and older in the 2012-2013 English Longitudinal Study of Ageing (ELSA). Frequent internet usage was found to be associated with lower levels of depression (affective well-being) and better quality of life (eudemonic well-being), but no association was found with life satisfaction (hedonic well-being) [21]. In contrast, Heo et al [22] found that more frequent internet use was associated with better life satisfaction and greater psychological well-being among HRS participants aged 65 years and older.

The current literature is limited on the purposes of internet use which have a positive (or negative) influence on mental health in older adults. The internet has myriad uses, for example, using the internet for communication purposes may improve mental health among older adults because it enables them to stay in touch with family and friends who previous generations may have lost contact with [23,24]. On the other hand, certain types of information access may undermine mental health. For example, using the internet for job searching was associated with more depressive symptoms, lower quality of life, and lower life satisfaction among ELSA participants [21]. Using the internet for finding new people and for entertainment was associated with decreased well-being among older Australians [25].

A possible confounder or moderator of the internet use and mental health association is socioeconomic position (SEP). Internet use is more prevalent in higher SEP groups [26-28], with use for information access and for entertainment being higher for persons with higher and lower SEP, respectively. SEP could also moderate this association; however, this has not been investigated in the literature to date. It could be the case that in groups with the highest levels of internet use (eg, the wealthiest and better educated), nonusers are at a greater disadvantage in terms of their mental health.

This study aimed to further the debate by using a longitudinal sample, analyzing the associations between internet usage and the hedonic dimension of well-being (life satisfaction) and the affective dimension of wellbeing (depression) over a 4-year period, analyzing different purposes of internet usage (eg, communication, entertainment, and information access), and examining whether SEP moderates these relationships. This study hypothesized that (1) more frequent internet use predicts better mental health longitudinally; (2) the association is moderated by SEP, being stronger in the higher SEP groups; and (3) mental health is most strongly associated with internet use for the purposes of communication (positively) and information access (negatively). Our findings will have implications for understanding the importance of internet use and its impact on mental health, which can inform policies to encourage wider and more frequent internet use in older adults to improve mental health.

## Methods

### Data and Sample

The ELSA is a nationally representative sample of the English population aged 50 years and older. Established in 2002, the sample has been followed up every 2 years [29]. Data were collected through computer-assisted personal interviews, self-completion questionnaires, and nurse assessments (every 4 years) [29]. The original sample was selected from the Health Survey for England (1998, 1999, and 2001) [30]. This analysis used data from waves 6 (2012-2013), 7 (2014-2015), and 8 (2016-2017). The analytical sample (n=9169 persons aged 50 years and older at wave 6, with 27,507 person-years of follow-up between waves 6 and 8) comprised sample members first recruited at waves 1 (n=5659), 3 (n=888), 4 (n=1796), and 6 (n=826). ELSA participants provided signed consent, and the London Multicentre Research Ethics Committee (MREC 01/2/91) granted ethical approval for the study.

### Time-Varying Outcomes

As an aspect of the affective dimension of hedonic well-being, mental health was measured using the validated 8-item version of the Center for Epidemiologic Studies Depression Scale (CES-D) [31]. Items were answered using a dichotomous response (*yes* or *no*), which were summed to result in a total score ranging from 0 to 8, where a score of 0 indicates no depressive symptoms. As an aspect of the cognitive dimension of hedonic well-being, life satisfaction was measured using the Satisfaction with Life Scale [32,33], which has been explicitly tested on older persons and consists of 5 items. The individual item responses ranged from *strongly agree* to *strongly disagree* on a 7-point Likert scale, which was summed to result in a total score ranging from 5 (lowest satisfaction) to 35 (highest satisfaction).

### Time-Varying Exposure

Data on the frequency of internet use were collected through self-completion questionnaires. The responses were recoded as *daily*, *weekly*, *monthly or less*, or *never*.

### Time-Constant Exposure

The purposes of internet use were assessed only for participants who reported using the internet at least once every 3 months. For this study, 12 purposes of internet use at wave 6 were grouped into 6 as follows: (1) communication (sending or receiving emails; use of social networking sites; and creating, uploading, or sharing content), (2) entertainment (news, newspaper or blog websites, streaming or downloading live or on demand television or radio, music, electronic books, and games), (3) information access (finding information about goods and services; searching for information for learning, research, and fact finding; and looking for a job or sending a job application), (4) managing finances, (5) electronic commerce (shopping and buying goods or services over the internet or selling goods or services over the internet), and (6) other. The questions on the purposes of internet use changed in waves 6, 7, and 8; therefore, we could not use it as a time-varying variable in the modeling. Our analyses focused on the 5 categories mentioned earlier.

### Time-Varying Covariates

Participants’ age was measured in a single year (coded to 99 to indicate those aged 91 years or older, whose individual age was not released). We tested nonlinear age and mental health associations using second-order polynomial terms. Couple status was measured according to whether a participant was cohabiting (married or unmarried) or not. Working status (currently working) was derived from economic activity status by dichotomizing those who were employees or self-employed from those who were seeking work, sick and not seeking work, retired, or unoccupied. Health status (presence of a limiting long-term illness) was measured through whether a participant self-reported an illness or disability that limited their activities in any way.

### Time-Invariant Covariates

SEP at wave 6 was measured using 3 different domains [34]: occupational class, wealth, and education. On the basis of degree of control and autonomy, occupational class (based on the household reference person’s last occupation) was measured using the 3-category version of the National Statistics Socio-Economic Classification: managerial and professional, intermediate, and routine and manual occupations [35]. Wealth was measured using quintiles of total net nonpension household wealth, a summary measure of the value of financial, physical, and housing wealth minus any debt [35]. It represents the permanent economic status of older adults [36], as most participants in aging cohort studies are retired. The highest educational qualification was grouped into 3 categories: degree level, below degree level, and no qualifications. Education among the ELSA cohort members is generally determined earlier in the life course, whereas wealth and occupational class are more likely to change during mid to later life.

### Statistical Analysis

To examine the longitudinal association between internet use and mental health, we used a series of fixed and random effects linear models. A nonlinear model for depression did not satisfy the condition of the fixed effects model, and the results from the random effects logistic regression model were substantively similar (not shown here). Therefore, the CES-D score was treated as a continuous dependent variable in both the fixed and random effects models. The fixed effects models control for all measured and unmeasured time-invariant characteristics by using each participant as their own control over survey waves. We adjusted for time-varying age, age squared, working status, couple status, and health status to address our first hypothesis.

H1: More frequent internet use predicts better mental health longitudinally.

Internet use was entered into the models as a four-category independent variable, with daily use as the reference. Random effects models do not control for the correlation between measured and unmeasured characteristics and, therefore, analyze both within- and between-participant variance. We adjusted for the same time-varying variables as for the fixed effects model and additionally adjusted for time-invariant SEP and sex to address our second hypothesis.

H2: SEP moderates the association between internet use and mental health.

An assessment of moderation was performed by including an interaction term between the frequency of internet usage and each marker of SEP; these were retained if statistically significant (likelihood ratio test, *P*<.10). Separate random effect models tested the association between the purpose of internet use and mental health to address our final hypothesis.

H3: There is a positive association between communication internet use and mental health and a negative association between information internet use and mental health.

The analysis not presented here showed similar results when limiting the analytical sample to those who used the internet compared with the results for all participants presented here.

To address attrition from wave 6 onward and item nonresponse within a survey wave, we fitted multiple imputation models by chained equations on all variables (outcomes, exposures, and covariates) with missing values. The purpose of internet use was imputed conditionally on whether a participant reported internet usage at wave 6. All statistical analyses were conducted using cross-sectional survey weights to minimize bias arising from sample attrition from the beginning of the ELSA study to participation in wave 6. The use of imputation and survey weights did not substantively alter the main findings.

## Results

### Descriptive Results

Table 1 presents the means, SDs, or percentages across person-waves for the key variables by frequency of internet usage. Overall, 56% of older adults used the internet daily, 12% weekly, 6% monthly or less, and 26% had never used the internet. The most common purpose was information access (68%), followed by communication (66%), electronic commerce (52%), entertainment (45%), and managing finances (39%). Those who used the internet more frequently had better life satisfaction scores; lower depression scores; and were more likely to be younger, currently working, be in a couple, have no limiting long-term illness, male, have a degree, be in managerial and professional occupations, and be in the wealthiest quintile compared with those who used the internet less frequently or not at all. For example, the mean age of a daily internet user was 66 (SD 7.64) years, whereas the mean age of a participant who had never used the internet was 75 (SD 9.30) years. Overall, 49% of daily users were male, compared with 38% of those who had never used the internet.

**Table 1:** Participant characteristics across person-waves by frequency of internet use (data are unweighted).

Measures		Daily	Weekly	Monthly	Never	Total
**Life satisfaction**						
	Mean (SD)^a^	26.12 (6.10)	25.10 (6.34)	24.44 (6.63)	24.72 (6.65)	25.56 (6.34)
	Participants, N	11,033	2294	1164	4847	19,338
**Depression**						
	Mean (SD)^a^	1.02 (1.61)	1.31 (1.77)	1.52 (1.95)	1.76 (2.04)	1.28 (1.80)
	Participants, N	11,136	2373	1203	5290	20,002
**Age (years)**						
	Mean (SD)^a^	65.81 (7.64)	68.66 (8.43)	69.64 (8.83)	74.86 (9.30)	68.78 (9.12)
	Participants, N	11,233	2388	1218	5349	20,188
**Currently working**						
	Participants, n (%)^a^	4304 (38.32)	629 (26.34)	281 (23.07)	519 (9.70)	5733 (28.40)
	Participants, N	11,233	2388	1218	5349	20,188
**In a couple**						
	Participants, n (%)^a^	8599 (76.55)	1686 (70.60)	846 (69.46)	2897 (54.16)	14,028 (69.49)
	Participants, N	11,233	2388	1218	5349	20,188
**Limiting long-term illness**						
	Participants, n (%)^a^	3058 (27.22)	832 (34.84)	484 (39.74)	2558 (47.82)	6932 (34.34)
	Participants, N	11,232	2387	1218	5347	20,184
**Internet purpose^b^**						
	Entertainment, n (%)	66.0	33.9	16.8	0	44.7
	Communication, n (%)	91.3	69.4	37.8	0	65.6
	Information access, n (%)	92.5	79.0	50.5	0	68.4
	Electronic commerce, n (%)	76.5	40.2	19.8	0	52.0
	Finances, n (%)	59.3	24.3	8.0	0	39.0
	Participants, N	10,627	2093	780	5349	18,849
**Sex^b^**						
	Female, n (%)	5772 (51.38)	1406 (58.88)	770 (63.22)	3314 (61.96)	11,262 (55.79)
	Participants, N	11,233	2388	1218	5349	20,188
**Education^b^**						
	Degree qualification, n (%)	3068 (27.31)	317 (13.27)	122 (10.02)	221 (4.13)	3728 (18.56)
	Below degree qualification, n (%)	6936 (61.75)	1590 (66.58)	809 (66.42)	2557 (47.80)	11,892 (59.19)
	No qualifications, n (%)	1183 (10.53)	469 (19.64)	285 (23.40)	2533 (47.35)	4470 (22.25)
	Participants, N	11,187	2376	1216	5311	20,090
**Occupational class^b^**						
	Managerial and professional, n (%)	5417 (48.22)	719 (30.11)	353 (28.98)	919 (17.18)	7408 (36.87)
	Intermediate, n (%)	2979 (26.52)	651 (27.26)	316 (25.94)	1277 (23.87)	5223 (26.00)
	Routine and manual, n (%)	2799 (24.92)	994 (41.62)	544 (44.66)	3138 (58.67)	7475 (37.21)
	Participants, N	11,195	2364	1213	5334	20,106
**Wealth quintile^b^**						
	Least affluent, n (%)	1046 (9.31)	302 (12.65)	194 (15.93)	1367 (25.56)	2909 (14.69)
	2, n (%)	1565 (13.93)	478 (20.02)	221 (18.14)	1272 (23.78)	3536 (17.86)
	3, n (%)	2063 (18.37	528 (22.11)	291 (23.89)	1329 (24.85)	4211 (21.27)
	4, n (%)	2809 (25.01)	581 (24.33)	284 (23.32)	830 (15.52)	4504 (22.75)
	Most affluent, n (%)	3484 (31.02)	461 (19.30)	206 (16.91)	490 (9.16)	4641 (23.44)
	Participants, N	10,967	2350	1196	5288	19,801

^a^Descriptive statistics for time-varying variables calculated using data from waves 6-8.

^b^Descriptive statistics for time-invariant variables calculated at wave 6 only.

### Frequency of Internet Use and Mental Health (Fixed Effects Models for Within-Person Change)

**Figure 1:**
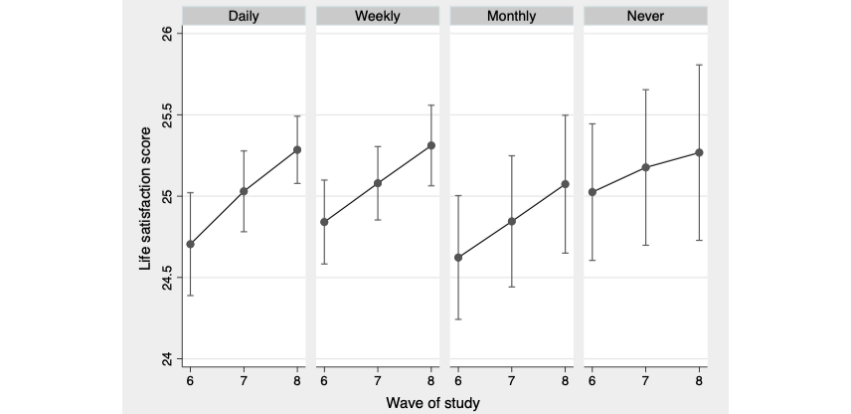
Predicted life satisfaction score by wave of study and frequency of internet usage from fixed effects model. Model adjusted for time-varying frequency of internet use, age, age square, working status, couple status, and health status.

Table 2 presents the fixed effects panel modeling estimates for the frequency of internet usage on depression and life satisfaction. The model estimates from the full models are shown in Multimedia Appendix 1. The coefficients represent estimates of change within participants between waves 6 and 8, and therefore positive estimates represent an increase in the depression score (a poorer health outcome) and an increase in the life satisfaction score (a positive health outcome). The frequency of internet use was not predictive of within-person change in depression when taking into account time-varying age, working, being in a couple, and health status. Daily internet usage did predict better life satisfaction over time compared with using the internet on, at most, a monthly basis when controlling for the same time-varying variables (β for monthly or less=−0.512; *P*=.02). On the basis of model estimates, Figure 1 shows that those using the internet daily were predicted to have more than a half-point increase in life satisfaction scores between waves 6 and 8. There was no significant within-person change in life satisfaction for weekly or never users (vs daily users).

**Table 2:** Fixed effects model coefficients for frequency of internet use on mental health outcomes.

Frequency of internet use^a^	Depression^b^		Life satisfaction^c^		
	Coefficient (95% CI)	*P* value		Coefficient (95% CI)	*P* value
Daily	Reference	N/A^d^		Reference	N/A
Weekly	0.030 (−0.822 to 0.143)	.59		−0.230 (−0.495 to 0.034)	.09
Monthly or less	0.111 (−0.060 to 0.281)	.20		−0.512 (−0.956 to −0.067)	.02
Never	0.096 (−0.101 to 0.293)	.33		−0.472 (−0.955 to 0.012)	.06

^a^Models adjusted for time-varying age, age squared, working status, couple status, and health status.

^b^Higher scores represent deteriorating depression within participants.

^c^Higher scores represent improving life satisfaction within participants.

^d^Not applicable.

### Socioeconomic Position as a Moderator of the Association Between Internet Use and Mental Health (Random Effects Models)

**Figure 2:**
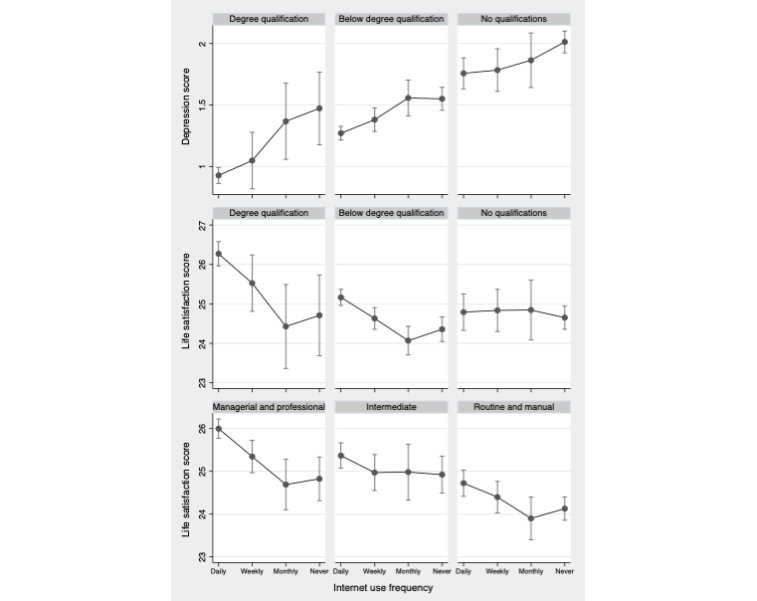
Predicted mental health score by socioeconomic position and frequency of internet usage from random effects model. Model adjusted for time-varying frequency of internet use, age, age square, working status, couple status, and health status and for time-invariant education, occupational class, wealth, and sex, including interaction terms for education and frequency of internet use and occupational class and frequency of internet use.

Figure 2 graphically illustrates the predictions from the random effects modeling, which showed that education and occupational class significantly moderated the longitudinal association between internet use frequency and mental health (likelihood ratio test *P*<.10 for interaction terms). The model estimates from the full models are presented in Multimedia Appendix 2. The differences in the predicted scores of depression (*P*=.09) and life satisfaction (*P*=.02) by internet use were steeper for those with a degree. For example, the predicted life satisfaction score for those who had no formal educational qualifications was almost 25 irrespective of internet use frequency, whereas the predicted score for those with a degree was over 1 unit higher for daily users compared with monthly or never users. Similarly, the models suggested that occupational class moderated the association between internet use frequency and life satisfaction (*P*=.05) but not depression. There was no evidence to suggest that wealth moderated the associations.

### Association Between Purpose of Internet Use and Mental Health (Random Effects Models)

Table 3 presents the estimates from the random effects model for mutually adjusted purposes of internet usage on mental health (full estimates are presented in Multimedia Appendix 3). Compared with those not using the internet for this stated purpose, using the internet for communication purposes was associated with a lower depression score between waves 6 and 8 (β=−0.24; *P*=.002) and a higher life satisfaction score (β=.97; *P*<.001) when controlling for time-varying and time-invariant variables. In contrast, using the internet for information access was associated with a worse life satisfaction score (β=−0.86; *P*=.001).

**Table 3:** Random effects model coefficients for the purpose of internet use on mental health outcomes.

Purpose of internet use^a,^^b^	Depression (95% CI)^c^			Life satisfaction (95% CI)^d^	
	Coefficient (95% CI)	*P* value	Log odds (95% CI)		*P* value
Entertainment	0.047 (−0.027 to 0.121)	.21	0.093 (−0.023 to 0.412)		.56
Communication	−0.242 (−0.391 to −0.092)	.002	0.968 (0.509-1.428)^e^		<.001
Information access	−0.034 (−1.344 to 0.105)	.62	−0.858 (−1.344 to −0.372)^e^		.001
Electronic commerce	−0.098 (−0.199 to 0.004)	.06	0.322 (−0.035 to 0.678)		.08
Finance	−0.073 (−0.153 to 0.007)	.07	0.210 (−0.110 to 0.531)		.19

^a^Models adjust for time-varying age, age squared, working status, couple status, and health status and for time-invariant socioeconomic position and sex.

^b^Reference category was not using the internet for each purpose.

^c^Higher scores represent deteriorating depression.

^d^Higher scores represent improving life satisfaction.

Analysis of more specific purposes (Multimedia Appendix 4) showed that using the internet for email was the only communication purpose associated with better mental health longitudinally and that using the internet for job searching was the only information access purpose associated with deteriorating mental health. Adjustment for more detailed working status categories did not change our findings, suggesting that using the internet for job searching was associated with lower life satisfaction longitudinally over and above being unemployed or out of work.

## Discussion

### Principal Findings

This study partially confirmed our first hypothesis (H1) that more frequent internet use improves mental health. After adjustment for time-varying confounding factors, there was a positive longitudinal effect of using the internet daily compared with monthly or less on life satisfaction. Internet use frequency was not associated longitudinally with depression. The second hypothesis (H2) that SEP moderates the association between internet usage frequency and mental health was confirmed. This association was stronger for those with an educational degree. There was also a stronger association in those in managerial occupations and professional occupations in life satisfaction. We confirmed our third hypothesis (H3) that using the internet for communication purposes (specifically use of email) was associated with better mental health and that using the internet for information access (specifically for job searching) was associated with worse mental health.

Existing studies have found mixed results regarding the association between internet use and mental health. Notwithstanding methodological differences, our findings are consistent with studies that found that frequent internet use can have benefits for older adults’ depression and life satisfaction [13,15,21,34]. The discrepancy between the findings could be because of methodological issues, such as measurement of mental health [18], and varying sample characteristics, sizes, and context [37]. Very few previous studies have been able to analyze internet use frequency and its relationship with mental health longitudinally. Our findings are consistent with the limited literature available [15,16].

This is the first study to our knowledge that explores SEP as a potential moderator of longitudinal associations between internet use and mental health among older adults. Several studies draw conclusions from sample sizes of fewer than 300 respondents and are not generalizable to middle- and older-aged adults. In contrast, the results of this study come from a large and nationally representative sample of persons aged 50 years and older. Furthermore, the range of questions on internet use allowed us to extend knowledge on the impact of different purposes of internet use on mental health.

Evidence from this study suggests that markers of SEP moderate the association between internet use and mental health; frequent usage in the highest educational and occupational groups had a larger positive impact on mental health than frequent use among those in the lowest groups. This might suggest that low usage in higher SEP groups through digital exclusion, for example, may be particularly problematic for mental health. Our findings, however, need to be viewed in light of our mental health outcomes. The measure of mental health used in our study is determined solely on the affective (depression) and cognitive (life satisfaction) aspects of hedonic well-being, which may not be fully indicative of overall mental health, which also includes eudemonic aspects such as a sense of purpose or control. We cannot rule out the possibility that internet use fulfills eudemonic needs among older adults in disadvantaged SEP groups. Future studies should use a broader measure of mental health to assess whether the longitudinal association between internet use and mental health varies significantly across SEP groups. Further work could also explore whether the relationship between internet use frequency and mental health is moderated by SEP in younger samples in whom almost all use the internet. One might posit that our finding is a cohort effect because later-born generations are expected to enter older age with a much higher prevalence of internet usage.

Our findings highlight heterogeneity among different purposes of internet use. Using the internet for communication through email was associated with better mental health. This provides empirical support for the importance of communication for positive mental health, as it allows individuals to maintain strong social ties and contacts, thereby reducing the negative impacts of social isolation and loneliness on mental health [7,15,38,39]. In contrast, using the internet for job searching was associated with higher depression and worse life satisfaction over time. This is comparable with previous analyses of ELSA data. This finding may indicate dissatisfaction with one’s current job or economic activity status rather than a direct effect of using the internet for this purpose [21]. Our results emphasize the importance of disaggregating different internet usage purposes, as internet use among older adults is not homogenous [40] and impacts different aspects of mental health in different ways.

### Policy Recommendations

Our findings point to clear policy implications, the most pressing of which is the need for interventions to encourage older adults to use the internet, so as to build and maintain strong social ties, thereby avoiding the damaging impacts of social isolation and loneliness on mental health. Strategies to promote and increase internet usage among older adults could include setting up public Wi-Fi in places frequently visited by older people and where usage might be low because of barriers to access and digital exclusion. Design of policy interventions to increase internet usage should consider the fact that nonusers of the internet in older age are not homogenous entities but are heterogeneous with respect to gender, age, SEP, household composition, and attitudes toward the internet [41]. It is also important to recognize that for many older people, intrinsic training in how to use the internet did not take place during their formal education or working life, and therefore, the barriers to access can be much more than about availability. Therefore, modifying any psychological barriers to active engagement with the internet is also important. More specifically, to promote internet use for communication, public policy makers should encourage private providers of social networking tools to create age-friendly interfaces. Hardware also needs to be appropriate for novice users and users with impairments such as poor eyesight. This requires manufacturers to be sensitive to these needs when designing products and making the accessibility settings easy to navigate to change the text size, for example.

### Limitations

Several limitations of this study need to be acknowledged. The 8-item CES-D administered in ELSA does not measure depression, rather symptomology, and its association with internet usage may be confounded by omitted variables not included or imperfectly measured in this study. These might include the diagnosis of mental health problems and access to mental health services. Further research should theorize the possible pathways between internet usage and mental health to minimize potential confounding. The measurement of our main exposure is also imperfect, as we were unable to disaggregate daily use of the internet (almost half of the sample) by the number of hours spent on the internet on a typical day. Evidence from other studies suggests that spending an excessive amount of time on the internet correlates with negative mental health in younger age samples [42]. We are not aware of any evidence of the relationship between very frequent internet usage and mental health in older age. A further limitation is the limited 4 years of follow-up. More data points are necessary to explore in detail how any change in the frequency and purposes for internet usage impact mental health trajectories.

A final limitation of our study is the potential bias in our estimates because of the selective nature of participation in aging cohort studies, such as ELSA. Our study sample included participants from the refreshment samples added at waves 3, 4, and 6 to ensure a nationally representative sample of persons aged 50 years and older living in private households at the time of wave 6 (2012-2013). Although our analyses used the appropriate weights available for use with the ELSA study and used multiple imputed data to reduce (wave and item) nonresponse bias, the healthier nature of surviving participants could have inevitably led to an underestimation of the internet use and mental health associations.

### Conclusions

In summary, internet use can serve as a channel for improving the mental health of older adults. With population aging and increases in the prevalence of poor mental health, promotion of (affordable) internet use and minimization of psychological barriers to digital engagement could potentially help to promote healthy living in older age.

## Appendix

Multimedia Appendix 1 Full fixed effects models results.

Multimedia Appendix 2 Full random effects models results for education by internet usage results.

Multimedia Appendix 3 Full random effects models results for broad purpose of use.

Multimedia Appendix 4 Random effects model fixed effect coefficients for detailed purpose of internet use on mental health.

## Abbreviations

CES-D: Center for Epidemiologic Studies Depression Scale
ELSA: English Longitudinal Study of Ageing
HRS: Health and Retirement Study
SEP: socioeconomic position
