# Supplementary material for: Exploring the Relationship Between Internet Use and Mental Health Among Older Adults in England: Longitudinal Observational Study
Source: J Med Internet Res. 2020 Jul 28;22(7):e15683. doi: 10.2196/15683 (PMC7420689; doi:10.2196/15683)
Supplement: Multimedia Appendix 1 [file jmir_v22i7e15683_app1.docx]

| **Table A1: Full fixed effects models results** | | |
| --- | --- | --- |
|  | **Depression (95% CI)^a^** | **Life Satisfaction (95% CI)^b^** |
| Daily | Reference | Reference |
| Weekly | 0.030 (-0.822, 0.143) | -0.230 (-0.495, 0.034) |
| Monthly or less | 0.111 (-0.060, 0.281) | -0.512 (-0.956, -0.067) |
| Never | 0.096 (-0.101, 0.293) | -0.472 (-0.955, 0.012) |
| Age | **-0.122 (-0.192, -0.054)** | **0.613 (0.388, 0.838)** |
| Age-squared | **0.001 (0.000, 0.001)** | **-0.004 (-0.005, -0.002)** |
| Coupled | **-0.669 (-0.893, -0.446)** | **1.499 (0.849, 2.148)** |
| Working | -0.083( -0.198, 0.032) | 0.175 (-0.227, 0.576) |
| Limiting illness | **0.181 (0.097, 0.265)** | **-0.437 (-0.663, -0.212)** |
| Constant | 6.157 (3.528, 8.786) | -0.214 (-8.855, 8.427) |
| *% within person σ^2^* | *60.4%* | *73.2%* |
| *Respondents* | *9,068* | *9,068* |
| *Respondent years* | *27,204* | *27,204* |
| *Notes.*  **^a^** Higher scores represent deteriorating depression within participants  **^b^** Higher scores represent improving life satisfaction within participants  Bold coefficients *P* < 0.001. | | |
