# Supplementary material for: Exploring the Relationship Between Internet Use and Mental Health Among Older Adults in England: Longitudinal Observational Study
Source: J Med Internet Res. 2020 Jul 28;22(7):e15683. doi: 10.2196/15683 (PMC7420689; doi:10.2196/15683)
Supplement: Multimedia Appendix 2 [file jmir_v22i7e15683_app2.docx]

| **Table A2: Full random effects models results for education by internet usage results** | | | |
| --- | --- | --- | --- |
|  | **Depression (95% CI)^a^** | **Life Satisfaction (95% CI)^b^** | |
|  | **Education by Internet use frequency** | **Education by Internet use frequency** | **Occupation by Internet use frequency** |
| **Fixed effects: time varying** | |  |  |
| Daily | Reference | Reference | Reference |
| Weekly | 0.121 (-0.120, 0.361) | -0.744 (-1.499, 0.011) | **-0.654 (-1.039, -0.268)** |
| Monthly or less | 0.440 (0.125, 0.755) | -1.843 (-2.973, -0.713) | **-1.308 (-1.926, -0.690)** |
| Never | **0.545 (0.247, 0.844)** | -1.560 (-2.665, -0.454) | **-1.173 (-1.707, -0.640)** |
| Age | **-0.134 (-0.172, -0.095)** | **0.464 (0.327, 0.602)** | **0.46 (0.323, 0.597)** |
| Age-squared | **0.001 (0.001, 0.001)** | **-0.003 (-0.004, -0.002)** | **-0.003 (-0.004, -0.002)** |
| Coupled | **-0.472 (-0.555, -0.389)** | **2.262 (1.970, 2.554)** | **2.26 (1.968, 2.552)** |
| Working | **-0.29 (-0.363, -0.217)** | 0.337 (0.039, 0.634) | 0.342 (0.044, 0.641) |
| Limiting illness | **0.646 (0.581, 0.711)** | **-1.268 (-1.473, -1.063)** | **-1.262 (-1.466, -1.058)** |
| **Fixed effect: time constant** | |  |  |
| Degree qualifications | Reference | Reference | Reference |
| Below degree | 0.084 (-0.003, 0.170) | -0.341 (-0.718, 0.036) | -0.237 (-0.573, 0.098) |
| No qualifications | 0.233 (0.076, 0.391) | -0.061 (-0.656, 0.535) | 0.525 (0.053, 0.997) |
| Managerial occupation | Reference | Reference | Reference |
| Intermediate occupation | 0.001 (-0.085, 0.086) | -0.230 (-0.546, 0.085) | -0.462 (-0.836, -0.087) |
| Routine occupation | 0.113 (0.030, 0.196) | -0.121 (-0.449, 0.206) | -0.306 (-0.724, 0.113) |
| Richest quintile | Reference | Reference | Reference |
| 2 | **-0.367 (-0.496, -0.237)** | **1.259 (0.809, 1.709)** | **1.256 (0.807, 1.705)** |
| 3 | **-0.599 (-0.729, -0.469)** | **1.965 (1.516, 2.415)** | **1.952 (1.504, 2.399)** |
| 4 | **-0.676 (-0.813, -0.539)** | **2.256 (1.801, 2.711)** | **2.252 (1.797, 2.708)** |
| Poorest quintile | **-0.72 (-0.86, -0.581)** | **3.368 (2.920, 3.816)** | **3.369 (2.920, 3.817)** |
| Female | **0.266 (0.203, 0.330)** | **0.290 (0.039, 0.541)** | **0.296 (0.044, 0.549)** |
| **Fixed effects: interaction** | |  |  |
| Weekly*below degree | -0.011 (-0.283, 0.261) | 0.211 (-0.627, 1.049) | - |
| Weekly*no quals | -0.094 (-0.383, 0.194) | 0.787 (-0.148, 1.723) | - |
| Monthly*below degree | -0.153 (-0.491, 0.185) | 0.748 (-0.474, 1.969) | - |
| Monthly*no quals | -0.334 (-0.758, 0.090) | 1.896 (0.420, 3.373) | - |
| Never*below degree | -0.266 (-0.591, 0.060) | 0.753 (-0.407, 1.912) | - |
| Never*no quals | -0.29 (-0.624, 0.043) | 1.420 (0.239, 2.600) | - |
| Weekly*intermediate | - | - | 0.257 (-0.317, 0.831) |
| Weekly*routine | - | - | 0.327 (-0.249, 0.904) |
| Monthly*intermediate | - | - | 0.924 (-0.061, 1.908) |
| Monthly *routine | - | - | 0.483 (-0.334, 1.301) |
| Never*intermediate | - | - | 0.728 (-0.013, 1.470) |
| Never*routine | - | - | 0.576 (-0.081, 1.233) |
| Constant |  |  | 3.786 (-1.236, 8.808) |
| *% within person σ^2^* | *50.6%* | *40.9%* | *40.9%* |
| *Respondents* | *9,169* | *9,169* | *9,169* |
| *Respondent years* | *27,507* | *27,507* | *27,507* |
| *Notes.*  **^a^** Higher scores represent deteriorating depression within participants  **^b^** Higher scores represent improving life satisfaction within participants  Bold coefficients *P* < 0.001. | | |  |
