# Supplementary material for: Exploring the Relationship Between Internet Use and Mental Health Among Older Adults in England: Longitudinal Observational Study
Source: J Med Internet Res. 2020 Jul 28;22(7):e15683. doi: 10.2196/15683 (PMC7420689; doi:10.2196/15683)
Supplement: Multimedia Appendix 3 [file jmir_v22i7e15683_app3.docx]

| **Table A3: Full random effects models results for broad purpose of use** | | |
| --- | --- | --- |
|  | Depression (95% CI)**^a^** | Life-Satisfaction (95% CI)**^b^** |
| **Fixed effects: time varying** | | |
| Entertainment | 0.047 (-0.027, 0.121) | 0.093 (-0.227, 0.412) |
| Communication | -0.242 (-0.391, -0.092) | **0.968 (0.509, 1.428)** |
| Information access | -0.034 (-0.174, 0.105) | **-0.858 (-1.344, -0.372)** |
| E-commerce | -0.098 (-0.200, 0.004) | 0.322 (-0.035, 0.678) |
| Finance | -0.073 (-0.153, 0.007) | 0.21 (-0.110, 0.531) |
| Age | **-0.141 (-0.180, -0.103)** | **0.468 (0.34, 0.596)** |
| Age-squared | **0.001 (0.001, 0.001)** | **-0.003 (-0.004, -0.002)** |
| Coupled | **-0.479 (-0.560, -0.397)** | **2.309 (2.000, 2.618)** |
| Working | **-0.296 (-0.378, -0.214)** | 0.351 (0.079, 0.622) |
| Limiting illness | **0.649 (0.589, 0.709)** | **-1.22 (-1.442, -0.997)** |
| **Fixed effects: time constant** | | |
| Degree qualifications | Reference | Reference |
| Below degree | 0.048 (-0.037, 0.132) | -0.25 (-0.597, 0.097) |
| No qualifications | 0.131 (0.018, 0.245) | 0.525 (0.094, 0.957) |
| Managerial occupation | Reference | Reference |
| Intermediate occupation | -0.009 (-0.089, 0.071) | -0.215 (-0.543, 0.113) |
| Routine occupation | 0.097 (0.016, 0.179) | -0.129 (-0.449, 0.192) |
| Richest quintile | Reference | Reference |
| 2 | **-0.353 (-0.481, -0.225)** | **1.196 (0.748, 1.645)** |
| 3 | **-0.577 (-0.700, -0.453)** | **1.847 (1.395, 2.299)** |
| 4 | **-0.651 (-0.778, -0.525)** | **2.154 (1.675, 2.633)** |
| Poorest quintile | **-0.685 (-0.81, -0.561)** | **3.299 (2.843, 3.755)** |
| Female | **0.261 (0.194, 0.329)** | 0.263 (0.005, 0.521) |
| Constant | 7.553 (6.100, 9.006) | 2.925 (-1.725, 7.575) |
| *% within person σ^2^* | *50.7%* | *41.0%* |
| *Respondents* | *9,169* | *9,169* |
| *Respondent years* | *27,507* | *27,507* |
| *Notes.*  **^a^** Higher scores represent deteriorating depression within participants  **^b^** Higher scores represent improving life satisfaction within participants  Bold coefficients *P* < 0.001. | | |
