# Supplementary material for: Exploring the Relationship Between Internet Use and Mental Health Among Older Adults in England: Longitudinal Observational Study
Source: J Med Internet Res. 2020 Jul 28;22(7):e15683. doi: 10.2196/15683 (PMC7420689; doi:10.2196/15683)
Supplement: Multimedia Appendix 4 [file jmir_v22i7e15683_app4.docx]

| **Table A4: Random effects model fixed effect coefficients for detailed purpose of internet use on mental health** | | |
| --- | --- | --- |
|  | **Depression (95% CI)^a^** | **Life Satisfaction (95% CI)^b^** |
| Email | **-0.22 (-0.347, -0.093)** | 0.609 (0.135, 1.082) |
| Find about goods and services | -0.105 (-0.251, 0.041) | 0.056 (-0.501, 0.613) |
| Search for information | -0.004 (-0.105, 0.096) | -0.345 (-0.700, 0.01) |
| Finance | -0.06 (-0.144, 0.024) | 0.166 (-0.159, 0.49) |
| Shopping | -0.073 (-0.165, 0.018) | 0.326 (-0.082, 0.735) |
| Selling | 0.093 (-0.05, 0.235) | -0.291 (-0.839, 0.257) |
| Social networking | 0.061 (-0.035, 0.157) | -0.269 (-0.623, 0.084) |
| Uploading content | 0.124 (-0.039, 0.286) | 0.144 (-0.434, 0.721) |
| News | -0.082 (-0.178, 0.015) | 0.212 (-0.116, 0.54) |
| Streaming | -0.036 (-0.125, 0.054) | 0.208 (-0.152, 0.567) |
| Games | 0.08 (-0.021, 0.181) | 0.015 (-0.349, 0.378) |
| Looking for job | 0.223 (0.059, 0.388) | **-1.293 (-1.978, -0.608)** |
| *Notes.*  **^a^** Higher scores represent deteriorating depression within participants  **^b^** Higher scores represent improving life satisfaction within participants  Bold coefficients *P* < 0.001. | | |
